# Human Activity Recognition for People with Knee Osteoarthritis—A Proof-of-Concept

**DOI:** 10.3390/s21103381

**Published:** 2021-05-12

**Authors:** Jay-Shian Tan, Behrouz Khabbaz Beheshti, Tara Binnie, Paul Davey, J. P. Caneiro, Peter Kent, Anne Smith, Peter O’Sullivan, Amity Campbell

**Affiliations:** 1 School of Allied Health, Faculty of Health Sciences, Curtin University, Perth 6845, Australia; Jay-Shian.Tan@curtin.edu.au (J.-S.T.); Tara.Binnie@curtin.edu.au (T.B.); P.Davey@curtin.edu.au (P.D.); JP.Caneiro@curtin.edu.au (J.P.C.); Anne.Smith@exchange.curtin.edu.au (A.S.); P.OSullivan@curtin.edu.au (P.O.); A.Campbell@curtin.edu.au (A.C.); 2 Curtin Institute for Computation, Curtin University, Perth 6845, Australia; Behrouz.Beheshti@curtin.edu.au

**Keywords:** knee osteoarthritis, machine learning, human activity recognition, inertial measurement units, physical activity monitoring

## Abstract

Clinicians lack objective means for monitoring if their knee osteoarthritis patients are improving outside of the clinic (e.g., at home). Previous human activity recognition (HAR) models using wearable sensor data have only used data from healthy people and such models are typically imprecise for people who have medical conditions affecting movement. HAR models designed for people with knee osteoarthritis have classified rehabilitation exercises but not the clinically relevant activities of transitioning from a chair, negotiating stairs and walking, which are commonly monitored for improvement during therapy for this condition. Therefore, it is unknown if a HAR model trained on data from people who have knee osteoarthritis can be accurate in classifying these three clinically relevant activities. Therefore, we collected inertial measurement unit (IMU) data from 18 participants with knee osteoarthritis and trained convolutional neural network models to identify chair, stairs and walking activities, and phases. The model accuracy was 85% at the first level of classification (activity), 89–97% at the second (direction of movement) and 60–67% at the third level (phase). This study is the first proof-of-concept that an accurate HAR system can be developed using IMU data from people with knee osteoarthritis to classify activities and phases of activities.

## 1. Introduction

Osteoarthritis is one of the leading causes of disability [1,2]. People with knee osteoarthritis have symptoms such as pain and stiffness that result in difficulty performing specific physical activities such as transitioning from a chair, negotiating stairs [3] and walking [4]. A recent review on the application of machine learning for people with knee osteoarthritis identified that movement-based (biomechanical) data has predominantly been used for the purposes of diagnosis and prediction of outcome in people with knee osteoarthritis [5]. While there is growing interest in wearable sensor technology for use in clinical environments, no studies have investigated if machine learning approaches can assist with *monitoring improvement* in the performance of clinically relevant activities outside of a clinical environment, for patients diagnosed with knee osteoarthritis [5]. 

There is high-quality evidence of improvements in pain and function following movement interventions, such as exercise, or surgical interventions. For people receiving these treatments, the leading medical society dedicated to researching osteoarthritis, the Osteoarthritis Research Society International, recommends that people who have a confirmed diagnosis of knee osteoarthritis are monitored for improvement in the performance of three specific and clinically relevant everyday activities; (1) transitioning from a chair, (2) negotiating stairs and (3) walking [6]. These movements are clinically relevant because they are related to pain, stiffness and reduced ability to participate in society. 

To assess if someone diagnosed with knee osteoarthritis is improving because of a treatment, clinicians are currently limited to assessing the performance of clinically relevant painful activities only in *observed* conditions such as in a clinic. Usually, a clinician would watch their patient perform the activity, however a single observation in a clinic does not demonstrate if a person is avoiding or doing less of this activity when *unobserved* after leaving the clinic. 

Currently, the best method of assessing unobserved activities is to use questionnaires known as Patient Reported Outcome Measures [6]. However, questionnaires can be unreliable because they assess a patient’s *perception* of their ability to perform an activity which does not objectively measure how many times or how *they actually perform* an activity when at home or at work. Wearable sensor technology has the potential to be used to help clinicians monitor the patient’s progress when they are *unobserved.*

One type of wearable sensor, inertial measurement units (IMUs), can collect movement-based information that can be processed into clinically relevant biomechanical data through fusion [7] or machine learning algorithms [8]. These methods have been reported to provide biomechanical outputs that are useful for clinicians, such as kinematics (e.g., knee flexion angle or knee angular velocity) [8,9,10] during a specific phase of an activity (e.g., stance phase of ascending stairs) when the patient is observed as part of an assessment in the clinic. 

IMUs enable data collection outside of the clinical environment and can be used when patients are *unobserved*. However, IMUs create large and unlabeled datasets making it difficult to identify which activities were performed, because the patient was unobserved. One approach to identifying when an activity or phase of an activity was performed from IMU data is through a machine learning approach known as human activity recognition (HAR). HAR models are built from algorithms to automate the process of classifying performance of human activities. As there is limited feasibility and practicality for clinicians to observe people with knee osteoarthritis in unobserved conditions, HAR has the potential to provide clinically relevant activity data from large continuous datasets. Having a system that can automatically label when an activity was performed could be subsequently used to monitor if a patient is improving by providing objective data about whether they are performing an activity more frequently, or to segment the data so that it can be used for subsequent biomechanical analysis. 

The majority of HAR models are built using traditional machine learning approaches (e.g., support vector machines, random forest, k-nearest neighbor); however, alternative approaches using deep neural networks such as convolutional neural networks (CNN) have recently demonstrated superior accuracy [11,12,13,14]. The benefit of a CNN model is that it automatically detects important features from input data, minimizing programming requirements typically required for traditional machine learning approaches. In addition, deep learning approaches like CNN are able to handle nonlinear interactions between features, something which is limited when using traditional machine learning approaches where features are defined by the researcher. There are many laboratory studies that have reported the accuracy of classifying physical activities from IMU data [12,13,14,15,16,17,18,19,20,21,22,23,24,25,26,27] using both traditional and deep learning approaches. Ramanujam et al. [28] provide a review of the most up-to-date computational advances in deep learning for HAR which is beyond the scope of this paper. 

There are multiple studies that have developed HAR models to classify daily activities using the lower limbs (e.g., standing, walking, going up stairs, walking down a hill), with accuracy ranging from 83% to 98% using training data from healthy people [12,13,14,15,16,18,20,25,26,27]. Other studies have classified specific phases of activities like transitioning from sit to stand or the stance phase of walking, reporting accuracy >99% [22,23]. In studies that have specifically developed HAR models for people who have knee osteoarthritis, models have been reported to classify a variety of rehabilitation exercises with accuracy >97% [19,21]. These studies are limited for two reasons.

The first limitation is that these models have been trained and tested on healthy, typically young, participants rather than people with knee osteoarthritis [14,16,19,21,22,23,25,26,27]. These HAR models have not yet been trained and tested on people who have been diagnosed with knee osteoarthritis. There is a substantial body of research demonstrating that movement characteristics of people who have knee osteoarthritis are significantly different from those of healthy people when performing activities like transitioning from a chair, negotiating stairs and walking [29,30,31,32], and that their movement is more variable [29,33,34]. Therefore, it is currently unknown if a HAR model could accurately classify activities from training data collected from people who have knee osteoarthritis. 

Previous studies have demonstrated that HAR models trained on data from healthy people are less accurate for use in people who have health conditions that affect how they move [35,36]. For example, using a support vector machine model trained on data from healthy people, Albert et al. [35] reported significantly less accuracy of their model when tested on people who have Parkinson’s disease (75%), compared to healthy participants (86%). In another study using a random forest classifier, Lonini et al. [36] reported 26% lower median accuracy for classification predicting five activities when training data using healthy participants was tested on people who use knee–ankle–foot orthoses due to lower limb impairments. Together, these studies suggest that HAR models trained on data from people who have abnormal movement characteristics because of medical conditions (e.g., knee osteoarthritis or Parkinson’s disease) result in poorer test accuracy in the patient population. 

The second limitation is that so far, studies that have developed HAR for people who have knee osteoarthritis have only trained and tested HAR models to classify rehabilitation exercises in healthy people [16,19,21] rather than functional activities such as walking, standing from a chair or using stairs, which are activities most important for clinicians to monitor for improvement after being prescribed exercises or after surgery. 

To date, no studies have addressed these two limitations when reporting the development and validation of a HAR model intended for use in people with knee osteoarthritis. Therefore, we aimed to develop a HAR system that could classify the activities and phases of transitioning from a chair, negotiating stairs and walking using raw IMU training data from people who have knee osteoarthritis using CNN models. In this study, we have demonstrated a proof-of-concept that data collected from people who have a confirmed diagnosis of knee osteoarthritis can feasibly be used to train a HAR model using CNN architecture to classify clinically relevant activities and phases of activities at an acceptable level of accuracy.

## 2. Materials and Methods

Eighteen participants with the clinical diagnosis of knee osteoarthritis [37] were recruited from local health provider clinics through direct referral and noticeboards. Inclusion criteria included ≥3 months of pain, ≥4/10 pain on most days and moderate activity limitation (a single item on the Function, Daily Living subscale of the Knee injury and Osteoarthritis Outcome Score) [38]. Exclusion criteria were previous lower limb arthroplasty, severe mobility impairments (e.g., neurological disorders, fracture) or an inability to complete the physical assessment due to language or cognitive difficulties. As soft tissue artefacts result in ‘noise’ in IMU data, to minimize the impact of this, participants were excluded if they had a body mass index (BMI) >30 kg/m^2^, or had relatively more soft tissue around the thigh with a waist-to-hip ratio (WHR) of ≤0.85 for women and ≤0.95 for men. All participants provided written informed consent and institutional ethics approval was obtained (HRE2017-0695) prior to data collection. The characteristics of participants are reported in Table 1.

### 2.1. Data Collection

Data were collected during a single session (average approximately 30 min) in a motion analysis laboratory. Height and weight data were collected using a manual stadiometer and calibrated digital scale prior to placing IMUs (v6 research sensors, DorsaVi, Melbourne, Australia) and retro-reflective markers on the participant. Participants then performed flexion–extension of the knee approximately 10 times as warm-up movements for both knees. A standardized battery of functional activities was performed by participants that included: transitioning from a chair (5 trials of sit-to-stand-to-sit), negotiating stairs (3 trials of a 3-stair ascent, 3 trials of a 3-stair descent) and walking (3 trials of a 5 m self-paced walk).

### 2.2. Activities for Classification

The levels of classification are outlined in Table 2. Three activities were classified at the first level, four at the second level and six at the third level.

### 2.3. Instrumentation

**Figure 1:**
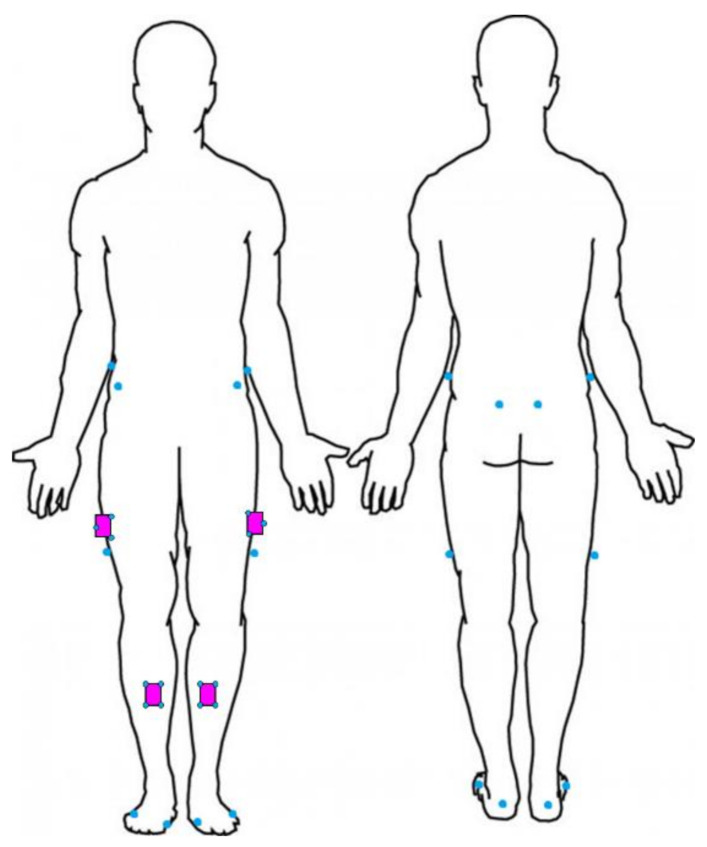
Placement of IMUs (purple) used for training the CNN models and Vicon marker (blue) placement for recording start and end times for each trial.

Two data collection systems were used. To train the HAR model, we used data from four DorsaVi IMUs placed on the thighs and shanks (Figure 1) of the participants. The location and number of sensors selected are the minimum number required to enable subsequent biomechanical analysis of both knees (e.g., knee joint flexion angle).

To precisely label the start and end times of each trial, we used a second system, an 18 camera Vicon three-dimensional motion analysis system (Oxford Metrics Inc., Oxford, UK). This was required as the IMU data were not able to be directly labeled while collecting data in the laboratory. Therefore, we time-synchronized the IMU and Vicon systems to allow labeling of the start and end of each trial in the IMU data. Synchronization procedures were performed by placing IMUs in a wooden box (with three retro-reflective markers attached) and rotated 10 times, >90° around the IMU’s X axis during a single Vicon data collection trial.

The sampling frequency for the IMU and Vicon systems were 100 and 250 Hz, respectively. IMUs were placed bilaterally on the lower limbs with double-sided hypoallergenic tape halfway between the superior edge of the greater trochanters and lateral epicondyles, halfway between the tibial tuberosities and anterior talocrural joints (Figure 1). Vicon retro-reflective markers were placed on anatomical landmarks of the pelvis and lower limb (Figure 1) consistent with previously published models which align with the International Society of Biomechanics recommendations [39].

### 2.4. HAR System Development

**Figure 2:**
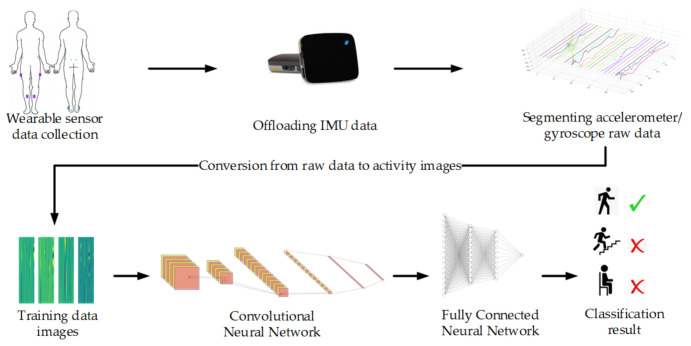
Architecture of the proposed human activity recognition system.

The architecture of the HAR system we developed is detailed through Section 2.4.1, Section 2.4.2 and Section 2.4.3 and summarized in Figure 2. Further details about the CNN and fully connected network are in Appendix A.

#### 2.4.1. Data Preparation

Raw IMU data (tri-axial accelerometer, gyroscope and magnetometer) were offloaded and output as time stamped files for each sensor. The reference standard kinematic data were processed in Vicon Nexus software (Oxford Metrics Inc., Oxford, UK). Reconstructed Vicon quaternion data and the filtered raw orientation data from each sensor’s accelerometer, gyroscope and magnetometer were time-synchronized by use of normalized cross-correlation using a customized LabVIEW program (National Instruments, Austin, TX, USA). Start and end times were exported for each activity for the raw IMU and reconstructed Vicon data. As there were a different number of samples for the stair phases (swing/stance), this resulted in an unbalanced dataset, which reduced model accuracy because of overfitting. Therefore, we balanced the dataset with an automated randomization procedure, whereby the dataset was shuffled each time and the number of selected samples was balanced to optimize the accuracy of the model. Magnetometer data were then discarded as it was not required for the development of the HAR model.

#### 2.4.2. Classification

One contemporary method for HAR model development is a machine learning approach known as deep learning. Deep learning uses a programmable neural network that automatically learns classification features from raw data, reducing the programming requirements used for other traditional machine learning methods. This approach automatically identifies complex features, rather than using predefined time and frequency domain features required for traditional machine learning HAR model approaches, such as support vector machines or k-nearest neighbor [40,41,42,43]. A convolutional neural network (CNN) is one type of deep learning approach that can be used for high dimensional time-series data [44] that outperforms traditional machine learning approaches [11,12,13,14]. 

**Figure 3:**
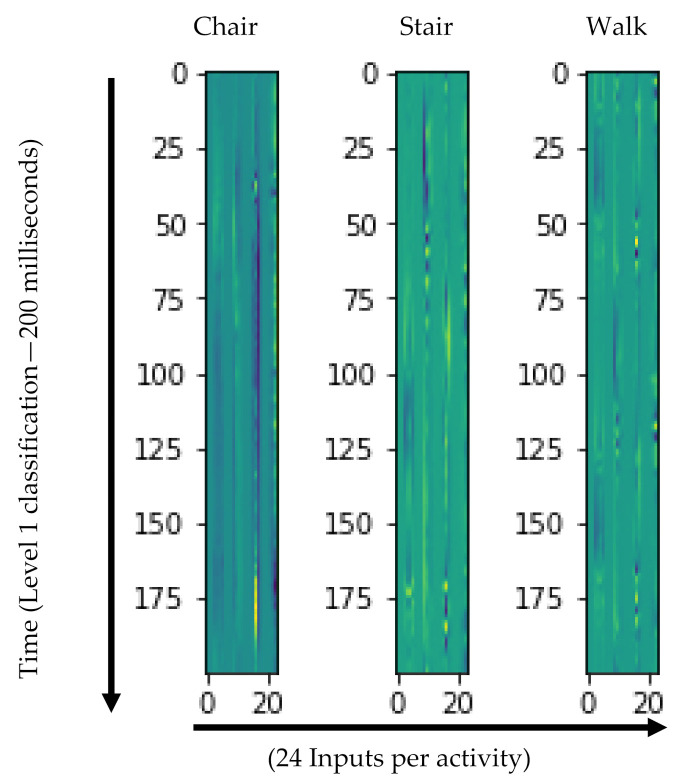
Visual representation of Level 1 activity ‘image’ patterns.

Deep neural networks such as CNNs are ideal for handling image data, like those from IMUs time-series data, which can be arranged into a two-dimensional ‘image’ as an input matrix. Features are then extracted automatically as each activity ideally represents a unique activity ‘image’ pattern (Figure 3). Input included triaxial (x, y, z) accelerometer and gyroscope data from all four IMUs resulting in a total of 24 inputs. Each input was stacked column by column then segmented into fixed size windows according to the level of classification. The images were then converted from a segmented numerical data array of the 12 accelerometer and 12 gyroscope inputs into images by normalizing the dataset to 0–255 range required for digital image production.

Appendix A depicts the CNN model architecture for each level of classification. Various architectures were tested using a different number of CNN layers, number of neurons in each layer, activation function, CNN kernel size, number of filters, max pooling size, number of dense layers in the fully connected part of the model, learning rate for ‘Adam’ optimization, and number of epochs. To avoid overfitting, we used cross-validation (see Section 2.5), data augmentation, adjusted the learning rate and used dropout and early stopping functions. Automatic feature extraction was performed at the first level of classification using two convolutional layers and three for the second and third level of classification. We developed a total of six models: one for level 1 (to classify between Chair, Stairs, and Walking), one for level 2-Chair (to classify between Sit down and Stand up), one for level 2-Stairs (to classify between Stairs ascending and Stairs descending), one for level 3-Stairs-Stairs ascending (to classify between Stance and Swing), one for level 3-Stairs-Stairs descending (to classify between Stance and Swing), and finally one for level 3-Walking (to classify between Stance and Swing). An optimization algorithm (Adam) trained the model [45] using different learning rates depending on the activity.

#### 2.4.3. Segmentation

**Figure 4:**
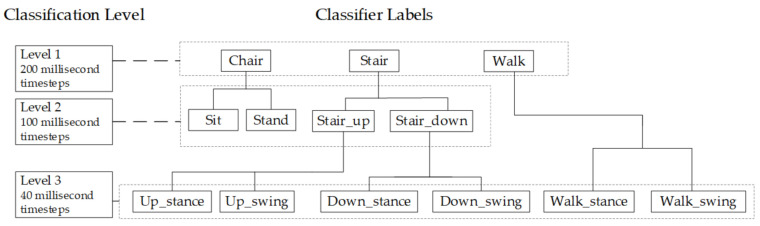
Decision tree for three levels of activity classification—Level 1 Activity, Level 2 Direction, Level 3 Phase.

A decision tree was developed (Figure 4), with a separate model created for each activity. 

**Figure 5:**
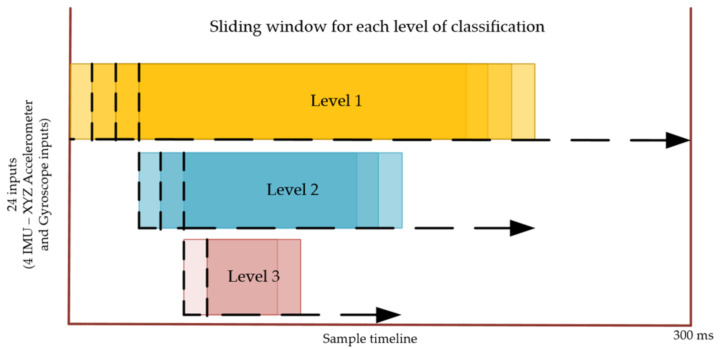
Illustration of the segmented window sliding in 10 ms increments for each level of classification. Level 1—200 ms; Level 2—100 ms; Level 3—40 ms.

IMU data were segmented at fixed window sizes of 200, 100 and 40 ms for each subsequent level of classification (Figure 5). Each of these windows slid 10 ms over the trial. The fixed window size was optimized to train the model by testing multiple window sizes for each level of classification. To determine the best window size, we created a distribution graph for all the trials and selected the window size based on the 80th percentile. An additional training image was produced for each additional 10 ms where the duration was longer than the fixed window size. For example, if the trial length was 240 ms, then there were five images created which allowed data augmentation. In situations where there was an overlapping window between two activities (e.g., Level 3 classification between swing and stance), the window was classified based on the higher prediction probability.

### 2.5. Model Performance—Statistical Testing

The accuracy of the models was evaluated using a leave-one-out cross-validation (LOOCV) method [46]. This method of validation trains the model on all participants except one and independently tests the model on the participant that is ‘left out’ (e.g., trained on 17, tested on one). Tests are repeated until each participant has been left out, and the reported result is the average across all participants. This model was chosen as it is more clinically relevant, as LOOCV provides an estimate that would more closely approximate the average accuracy for individual patients than other validation approaches [46]. We evaluated the accuracy (1), precision (2) and recall (3) of our model. In addition, we present a confusion matrix which depicts the total of the binary (correct/incorrect) classifications from LOOCV across all participants.

(1) $$Accuracy=\frac{TP+TN}{TP+FP+TN+FN}$$

(2) $$Precision=\frac{TP}{TP+FP}$$

(3) $$Recall=\frac{TP}{TP+FN}$$

## 3. Results

**Figure 6:**
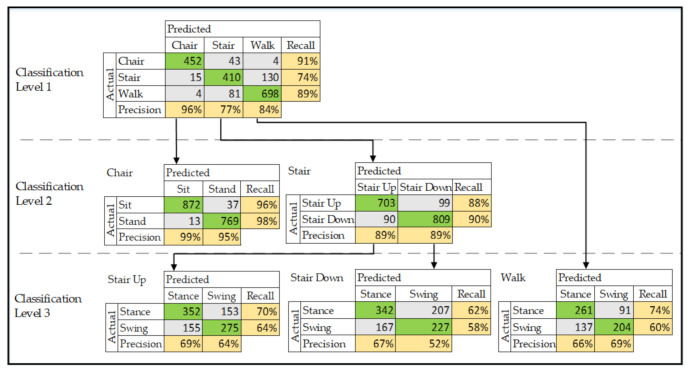
Confusion matrices for classification of activities/phases per classification level. Green cells represent correct classification and arrows represent the classification pathway from activities to phases of activities.

The overall accuracy across multiple levels of classification ranged from 60% to 97% (Table 3). Confusion matrices are presented in Figure 6 for each level of classification.

## 4. Discussion

Previous literature reporting the development of HAR models for people with knee osteoarthritis had not explored (1) the potential accuracy of a model trained on data collected from people who have knee osteoarthritis rather than healthy people, and (2) the capacity of such a model to classify activities related to the disability experienced by people with knee osteoarthritis rather than rehabilitation exercises. Therefore, the aim of this proof-of-concept study was to use IMU data collected from people who have knee osteoarthritis to train a HAR system to classify clinically relevant activities and phases of those activities.

While many previous HAR studies have investigated novel computational methods to optimize HAR models, we took a different approach and demonstrated two novel findings, that (1) a HAR model can be trained on IMU data collected from participants who have knee osteoarthritis rather than healthy people, and (2) activities of transitioning from a chair, negotiating stairs and walking can be classified from training data collected from this specific population. The model accuracy was 85% at the first level of classification, 89–97% at the second and 60–67% at the third. Our model performed with a high degree of accuracy compared to studies using the same validation approach (LOOCV). 

### 4.1. Comparison of HAR System Accuracy to Previous Literature

The results of our HAR model (accuracy range 60–97%) are consistent with previous studies that classified activities of the lower limb in healthy people and people with medical conditions that affect their movement (e.g., Parkinson’s disease) which have reported model accuracy between 75% and 99% [14,25,26,27,35,36,46,47,48]. These previous studies use a single HAR model to classify between 5 and 12 activities resulting in an overall accuracy for the single model. As our HAR system used a decision tree framework to classify both activities and phases of those activities for clinical purposes that are described in Section 4.4, comparison of our model is limited because we developed multiple HAR models for three levels of classification. Those three levels resulted in a total of six models with accuracy reported for each separate model (Figure 6). However, and despite this, the accuracy for the first and second level of classification (85–97%) are promising compared to previous studies that used a single model. 

At subsequent levels of classification, our model’s accuracy slightly improved at the second level of classification (range 89–97%), but at the third level of classification, swing and stance phases for stairs and walking, the accuracy (range 60–67%) was reduced. Usually, the accuracy of HAR algorithms reduces for finer levels of classification as predictions become more complex. Our results are consistent with these previous studies, where reductions in accuracy for subsequent level classification have been reported to range between 4.2% and 16.4% [20,24]. While we compared the results of our models to previous HAR literature, the considerable heterogeneity between these studies reduces the capacity to make meaningful comparisons [16]. This heterogeneity arises from a wide variety of factors that include: the type of activities to be classified, the number and location of sensors, the number of activities, the number of training data samples (and participants), the population sampled (e.g., healthy, knee osteoarthritis, Parkinson’s disease) and the validation approach (e.g., LOOCV or k-fold cross-validation).

The model was more accurate when classifying activities where movement patterns are distinct. For example, at the first level of classification, a chair transition was most frequently classified correctly (recall 91%—452 correct predictions from 499 observations). We believe the higher accuracy for a chair transition is due to a unique activity pattern where both legs perform synchronized movements. On the other hand, negotiating stairs was misclassified as walking 23% of the time (130 incorrect predictions from 555 observations). As walking and negotiating stairs share similar features of a reciprocal movement pattern where one leg is swinging forward while the other is in stance moving backwards alternating in a rhythmical manner, misclassification between these two activities is commonly reported in studies that trained HAR models using data from healthy participants [13,14,26]. One solution recently reported is to combine neural network models to classify eight activities that included walking, walking uphill, walking down hill, ascending stairs, descending stairs and running—all activities that share a reciprocal pattern [26]. Combining CNN with another deep learning approach known as long short-term memory resulted in superior model performance with fewer misclassifications for activities that have reciprocal patterns compared to an independent CNN or long short-term memory models alone.

### 4.2. Appropriate Validation Approaches for Clinical Populations

The most important consideration when comparing the accuracy of a HAR model is the validation approach. For IMU HAR systems to be widely adopted and accepted by healthcare clinicians and researchers, the validation approach described in the machine learning field is very important. It is important for a clinician to know the average error that exists for individual patients. For any particular patient where new data will be tested against the model, clinicians can have greater confidence in the accuracy of models that are validated with LOOCV rather than other validation approaches (such as k-fold or 70:30 cross-validation) [46].

Methods other than LOOCV inflate the accuracy of models designed to be used on a single individual. For example, one study [49] aggregated multiple HAR datasets that represented multiple machine learning models, sensor types and placements that included ambulatory activities such as walking, ascending and descending stairs, and jogging, amongst others. They reported an accuracy of 96.4% when using a 10-fold cross-validation compared with a 79.9% using LOOCV, representing a substantial 16.5% difference between these validation methods [49]. Similarly, in a HAR model that included walking and multiple stationary activities in a sample of participants with Parkinson’s disease, the accuracy of a support vector machine classifier reduced from 92.2% using a 10-fold cross-validation to 75.1% when using a LOOCV [35]. Therefore, in comparison, the accuracy of our CNN models ranging from 85% to 97% at the first and second level of classification using LOOCV is promising.

Although LOOCV has lower reported accuracy than some other validation approaches, it is preferred as it accounts for between-participant variability [35,46,49] which is important for a clinician who needs to know the average accuracy of a model as it applies to each new individual patient. 

### 4.3. The Importance of Representative Sampling

Validating a model for populations who have movement impairments related to medical conditions is particularly important because they move differently from healthy people. In the introduction of this paper, we provided a detailed description of the importance of training HAR models on data collected from people with medical conditions that affect their movement rather than healthy people. Briefly, previous studies have demonstrated that HAR models trained on data from healthy people are less accurate when tested on data collected from people with medical conditions that affect the way they move, reducing the model accuracy by up to 28% [35,36]. 

### 4.4. Clinical Application of HAR

A central component of an initial clinical interaction is to establish a diagnosis. After a person is diagnosed with knee osteoarthritis, they should be referred for core interventions like movement rehabilitation or surgery because both these treatments are helpful to improve pain and ability to perform activities like transitioning from a chair, negotiating stairs and walking. However, currently clinicians do not have an objective method to use outside of the clinical environment to monitor if people are improving after treatment. Therefore, a HAR system that can classify both clinically relevant functional activities and phases of those activities is potentially important for a clinician because it could help provide information about whether a person who has knee osteoarthritis is improving when outside of the clinic such as when at home or at work.

There are two potential ways a HAR system could be used for clinical purposes for a person with knee osteoarthritis when they are *unobserved* (at home or at work) while under the care of a clinician. Firstly, for the purposes of *physical activity monitoring*. For instance, one patient with knee osteoarthritis may avoid using the stairs due to a fear of falling. The clinician’s goal may be to increase use of stairs and therefore they could use a HAR system to automatically and objectively count the number of times their patient used stairs during a period of physical activity monitoring. This is especially important as patient self-report of physical activity does not consistently correlate with wearable sensor-based monitoring [50,51]. 

Secondly, a HAR-IMU system could be used to segment the data for subsequent *biomechanical analysis of activity phases for data collected when unobserved*, outside of the clinic. Currently, most IMU systems can only be used in *observed* conditions where the start and end times of a data capture trial are known. Under *unobserved* conditions, for instance when a person is at home or at work, IMU data are unlabeled which currently requires a clinician to process long, continuous datasets which is time-consuming and therefore not feasible. HAR provides a solution to segment the data for unobserved biomechanical analysis. For instance, a patient may have specific difficulties with the stance phase of descending stairs because of their stiff knee. The clinician’s goal in this situation may be to change specific biomechanical patterns with the aim of reducing knee stiffness during this activity. In this case, the clinician may use HAR to identify instead a window in the IMU data when the stance phase occurred when walking down stairs for subsequent biomechanical analysis, to monitor if their patient can bend their knee more when going down stairs after they receive treatment. Therefore, a HAR system that can automatically segment the data by labeling the start and end times of a phase of an activity, when the patient is unobserved, could help a clinician monitor improvement of a specific biomechanical parameter (e.g., knee joint angle or force).

### 4.5. Clinician and Patient Burden

It is important that HAR model development considers both the intended population that will wear the sensors (e.g., a patient with knee osteoarthritis) as well as the intended population that will use that information (e.g., a clinician). Therefore, the choice as to the number of IMUs should balance the patient burden of wearing multiple sensors with optimizing the accuracy of the model and any potential clinical benefit that may ensue. We chose to use a total of four IMUs on each participant, with two sensors on each lower limb for two reasons. 

Firstly, studies have demonstrated higher accuracy with more sensors, especially across multiple body regions [20,52]. For example, using a 10-fold cross-validation, Lee et al. [52] demonstrated a reduction in HAR model accuracy of up to 8% when using inputs from three, rather than five IMUs when classifying six different squatting tasks [49]. Secondly, a sensor placed on both the thigh and the shank is required for subsequent biomechanical analysis of the knee using current IMU fusion algorithms. We therefore believe that four IMUs strikes a balance between optimal accuracy and participant burden while providing clinically relevant information for knowing if a patient is improving during treatment. 

Studies have determined the best location for IMU placement that optimizes model accuracy from as few as one sensor in healthy people [49]. However, optimal sensor positions could differ in healthy people compared to those with medical conditions that affect their movement. Additionally, the purpose of the HAR model needs to be considered, especially if biomechanical analysis is required which requires at least two sensors to estimate the biomechanical parameters around a joint like the knee. Future HAR model development for people who have medical conditions that affect their movement, such as people who have knee osteoarthritis, should carefully consider IMU placement for the two clinical purposes of (1) identifying activities to count the frequency or duration of performance or alternatively (2) as a means of segmenting data to capture a window of activity that could be used for subsequent biomechanical analysis.

### 4.6. Strengths, Limitations, and Future Research

This study is the first to describe the development of a HAR model that (1) used training data collected from people who have knee osteoarthritis, (2) includes activities that are recommended by medical guidelines to monitor improvement in this population and (3) can identify not only activities but also phases of activities useful for biomechanical analysis. The training data for our HAR model did not include a diverse range of participants, such as those who are severely disabled, obese, or have substantially different patterns of movement related to pain such as a ‘step-to’ gait pattern when using stairs. Future studies should include these diverse patient presentations to allow greater generalizability of a model.

Validation of this IMU-based HAR model is required using data collected in conditions outside a laboratory environment (e.g., clinic or home). With further development, this model could be used in a workflow for analyzing data collected when a patient was unobserved in order to segment data for subsequent biomechanical analysis using IMU fusion algorithms or machine learning predictions for knee joint kinematics (angles and speed) and kinetics (force or moments).

While the accuracy of our models was high for the first and second levels of classification, the accuracy for the third level of classification was substantially less. Subsequent studies should explore how to optimize model accuracy over multiple levels of classification.

Most of the published HAR models have limited capacity to be used in unobserved conditions as people perform many diverse activities other than those on which the model was trained. The current model may therefore produce a significant number of false positive classifications in unobserved conditions. Future HAR model development trained on data collected in laboratory conditions should also be validated for use in less controlled environments such as a clinic or in a person’s home. The investigation of approaches that combine CNN with other machine learning approaches (e.g., CNN long short-term memory) is recommended as these approaches may further improve classification accuracy for activities that share similar features. Further investigation is warranted to explore the best number, location and combination of sensors in the population with knee osteoarthritis.

## 5. Conclusions

Our results provide a proof-of-concept that data collected from people with knee osteoarthritis can be used to train HAR models to classify clinically relevant activities, and phases of those activities that could be used for the purpose of monitoring an improvement due to treatment. This is the first study to develop a Human Activity Recognition (HAR) model from data collected from people who have knee osteoarthritis to classify clinically relevant activities and phases of activities in this population. The model accuracy was 85% at the first level of classification, 89–97% at the second level of classification and 60–67% at the third level of classification. The performance of our models compares well to other studies that classified different activities using the same validation approach (LOOCV).

As we have demonstrated that these activities can be classified in the population, it may be possible to develop a HAR system that can objectively measure the number of times a person who has knee osteoarthritis performs an activity, as well as segment data to allow biomechanical analysis of these activities for data collected at home or at work. 

## Figures and Tables

**Table 1:** Characteristics of participants.

	All Participants (*n* = 18)		
Characteristics	Mean	SD	Range
Age (yr)	66.2	8.7	49–82
Female (%)	53%		
Weight (kg)	80.5	15.9	44–113
Height (m)	1.7	0.1	157–186.5
BMI (kg/m^2^)	26.6	15.9	17.8–33.4

**Table 2:** Levels of classification.

Level 1	Level 2	Level 3
Chair	Sit down	
	Stand up	
Stairs	Stairs ascending	Stance
		Swing
	Stairs descending	Stance
		Swing
Walking		Stance
		Swing

**Table 3:** Accuracy of CNN models using leave-one-out cross-validation for each level of classification.

	Accuracy
1st Level Classification	
Chair, Stair, Walk	85%
	
2nd Level Classification	
Chair—stand/sit	97%
Stair—up/down	89%
	
3rd Level Classification	
Stair up stance/swing	67%
Stair down stance/swing	60%
Walk stance/swing	67%

## Appendix A

**Table A1:** Classification Level 1—Chair, Stair, Walk.

	Learning Rate: 0.0001									
	Convolution				Transition	Fully Connected				
**Type**	CONV2D	MAXPOOLING2D	CONV2D	MAXPOOLING2D	Flatten	Dense	Activation	Dropout	Dense	Activation
**Filter size**	32		64							
**Kernel size**	3 × 3	2 × 2	3 × 3	2 × 2						
**Function**							ReLu			Softmax
**Dropout size**								0.2		
**Neurons**						100			3	

**Table A2:** Classification Level 2—Stair Up/Down, Chair Sit/Stand.

	Learning Rate: Chair 0.0001, Stair 0.001														
	Convolution									Transition	Fully Connected				
**Type**	CONV2D	Activation	MAX POOLING2D	CONV2D	Activation	MAX POOLING2D	CONV2D	Activation	MAX POOLING2D	Flatten	Dense	Activation	Dropout	Dense	Activation
**Filter size**	32			64			64								
**Kernel size**	3 × 3		2 × 2	3 × 3		2 × 2	3 × 3		2 × 2						
**Function**		ReLu			ReLu			ReLu				ReLu			Softmax
**Neurons**											100			2	

**Table A3:** Classification Level 3—Walk Swing/Stance and Stair Up/Down Swing/Stance.

	Learning Rate: Walk/Stair Down 0.001, Stair Up 0.0001													
	Convolution								Transition	Fully Connected				
**Type**	CONV2D	Activation	MAXPOOLING2D	CONV2D	Activation	CONV2D	Activation	MAXPOOLING2D	Flatten	Dense	Activation	Dropout	Dense	Activation
**Filter size**	32			64		64								
**Kernel size**	3 × 3		2 × 2	3 × 3		3 × 3		2 × 2						
**Function**		ReLu			ReLu		ReLu				ReLu			Softmax
**Neurons**										100			2	

## References

[1] Ackerman I.N., Bohensky M.A., Zomer E., Tacey M., Gorelik A., Brand C.A., de Steiger R. (2019). The projected burden of primary total knee and hip replacement for osteoarthritis in Australia to the year 2030. BMC Musculoskelet. Disord..

[2] Vos T., Barber R.M., Bell B., Bertozzi-Villa A., Biryukov S., Bolliger I., Charlson F., Davis A., Degenhardt L., Dicker D. (2015). Global, regional, and national incidence, prevalence, and years lived with disability for 301 acute and chronic diseases and injuries in 188 countries, 1990–2013: A systematic analysis for the Global Burden of Disease Study 2013. Lancet.

[3] Machado G.P.M., Gignac M.A.M., Badley E.M. (2008). Participation restrictions among older adults with osteoarthritis: A mediated model of physical symptoms, activity limitations, and depression. Arthritis Care Res..

[4] Wilkie R., Peat G., Thomas E., Croft P. (2007). Factors associated with restricted mobility outside the home in community-dwelling adults ages fifty years and older with knee pain: An. example of use of the International Classification of Functioning to investigate participation restriction. Arthritis Care Res..

[5] Kokkotis C., Moustakidis S., Papageorgiou E., Giakas G., Tsaopoulos D.E. (2020). Machine learning in knee osteoarthritis: A review. Osteoarthr. Cartil. Open.

[6] Dobson F., Hinman R.S., Roos E.M., Abbott J.H., Stratford P., Davis A.M., Buchbinder R., Snyder-Mackler L., Henrotin Y., Thumboo J. (2013). OARSI recommended performance-based tests to assess physical function in people diagnosed with hip or knee osteoarthritis. Osteoarthr. Cartil..

[7] Weygers I., Kok M., Konings M., Hallez H., De Vroey H., Claeys K. (2020). Inertial sensor-based lower limb joint kinematics: A methodological systematic review. Sensors.

[8] Mundt M., Koeppe A., David S., Witter T., Bamer F., Potthast W., Markert B. (2020). Estimation of gait mechanics based on simulated and measured IMU data using an artificial neural network. Front. Bioengi. Biotechnol..

[9] Drapeaux A., Carlson K. (2020). A comparison of inertial motion capture systems: DorsaVi and Xsens. Int. J. Kinesiol. Sports Sci..

[10] Van der Straaten R., De Baets L., Jonkers I., Timmermans A. (2018). Mobile assessment of the lower limb kinematics in healthy persons and in persons with degenerative knee disorders: A systematic review. Gait Posture.

[11] Brock H., Ohgi Y., Lee J. (2017). Learning to Judge Like a Human: Convolutional Networks for Classification of Ski Jumping Errors. Proceedings of the 2017 ACM International Symposium on Wearable Computers.

[12] Jiang W., Yin Z. (2015). Human Activity Recognition Using Wearable Sensors by Deep Convolutional Neural Networks. Proceedings of the 23rd ACM International Conference on Multimedia.

[13] Chen Y., Xue Y. A Deep Learning Approach to Human Activity Recognition Based on Single Accelerometer. Proceedings of the 2015 IEEE International Conference on Systems, Man, and Cybernetics.

[14] Fridriksdottir E., Bonomi A.G. (2020). Accelerometer-based human activity recognition for patient monitoring using a deep neural network. Sensors.

[15] Cust E.E., Sweeting A.J., Ball K., Robertson S. (2019). Machine and deep learning for sport-specific movement recognition: A systematic review of model development and performance. J. Sports Sci..

[16] O’Reilly M., Caulfield B., Ward T., Johnston W., Doherty C. (2018). Wearable inertial sensor systems for lower limb exercise detection and evaluation: A systematic review. Sports Med..

[17] Rast F.M., Labruyère R. (2020). Systematic review on the application of wearable inertial sensors to quantify everyday life motor activity in people with mobility impairments. J. NeuroEng. Rehabil..

[18] Charlton P.C., Kenneally-Dabrowski C., Sheppard J., Spratford W. (2017). A simple method for quantifying jump loads in volleyball athletes. J. Sci. Med. Sport.

[19] Chen K.-H., Chen P.-C., Liu K.-C., Chan C.-T. (2015). Wearable sensor-based rehabilitation exercise assessment for knee osteoarthritis. Sensors.

[20] Hendry D., Chai K., Campbell A., Hopper L., O’Sullivan P., Straker L. (2020). Development of a human activity recognition system for ballet tasks. Sports Med. Open.

[21] Huang P., Liu K., Hsieh C., Chan C. Human Motion Identification for Rehabilitation Exercise Assessment of Knee Osteoarthritis. Proceedings of the 2017 International Conference on Applied System Innovation (ICASI).

[22] Martinez-Hernandez U., Dehghani-Sanij A.A. (2018). Adaptive Bayesian inference system for recognition of walking activities and prediction of gait events using wearable sensors. Neural Netw..

[23] Martinez-Hernandez U., Dehghani-Sanij A.A. (2019). Probabilistic identification of sit-to-stand and stand-to-sit with a wearable sensor. Pattern Recognit. Lett..

[24] Whiteside D., Cant O., Connolly M., Reid M. (2017). Monitoring hitting load in tennis using inertial sensors and machine learning. Int. J. Sports Physiol. Perform..

[25] Arif M., Kattan A. (2015). Physical activities monitoring using wearable acceleration sensors attached to the body. PLoS ONE.

[26] Ascioglu G., Senol Y. (2020). Design of a wearable wireless multi-sensor monitoring system and application for activity recognition using deep learning. IEEE Access.

[27] Emmerzaal J., De Brabandere A., Vanrompay Y., Vranken J., Storms V., De Baets L., Corten K., Davis J., Jonkers I., Vanwanseele B. (2020). Towards the monitoring of functional status in a free-living environment for people with hip or knee osteoarthritis: Design and evaluation of the JOLO blended care app. Sensors.

[28] Ramanujam E., Perumal T., Padmavathi S. (2021). Human activity recognition with smartphone and wearable sensors using deep learning techniques: A review. IEEE Sens. J..

[29] Astephen J.L., Deluzio K.J., Caldwell G.E., Dunbar M.J., Hubley-Kozey C.L. (2008). Gait and neuromuscular pattern changes are associated with differences in knee osteoarthritis severity levels. J. Biomech..

[30] Iijima H., Shimoura K., Aoyama T., Takahashi M. (2018). Biomechanical characteristics of stair ambulation in patients with knee OA: A systematic review with meta-analysis toward a better definition of clinical hallmarks. Gait Posture.

[31] Turcot K., Armand S., Fritschy D., Hoffmeyer P., Suvà D. (2012). Sit-to-stand alterations in advanced knee osteoarthritis. Gait Posture.

[32] Baliunas A.J., Hurwitz D.E., Ryals A.B., Karrar A., Case J.P., Block J.A., Andriacchi T.P. (2002). Increased knee joint loads during walking are present in subjects with knee osteoarthritis. Osteoarthr. Cartil..

[33] Gustafson J.A., Robinson M.E., Fitzgerald G.K., Tashman S., Farrokhi S. (2015). Knee motion variability in patients with knee osteoarthritis: The effect of self-reported instability. Clin. Biomech..

[34] Kiss R.M. (2011). Effect of severity of knee osteoarthritis on the variability of gait parameters. J. Electromyogr. Kinesiol..

[35] Albert M., Toledo S., Shapiro M., Koerding K. (2012). Using mobile phones for activity recognition in Parkinson’s patients. Front. Neurol..

[36] Lonini L., Gupta A., Kording K., Jayaraman A. Activity Recognition in Patients with Lower Limb Impairments: Do we need training data from each patient?. Proceedings of the 2016 38th Annual International Conference of the IEEE Engineering in Medicine and Biology Society (EMBC).

[37] National Clinical Guideline (2014). National Clinical Guideline. National Institute for Health and Clinical Excellence: Guidance. Osteoarthritis: Care and Management in Adults.

[38] Roos E.M., Lohmander L.S. (2003). The knee injury and osteoarthritis outcome score (KOOS): From joint injury to osteoarthritis. Health Qual. Life Outcomes.

[39] Wu G., Siegler S., Allard P., Kirtley C., Leardini A., Rosenbaum D., Whittle M., D’Lima D.D., Cristofolini L., Witte H. (2002). ISB recommendation on definitions of joint coordinate system of various joints for the reporting of human joint motion—Part I: Ankle, hip, and spine. J. Biomech..

[40] Hou C. A Study on IMU-Based Human Activity Recognition Using Deep Learning and Traditional Machine Learning. Proceedings of the 2020 5th International Conference on Computer and Communication Systems (ICCCS).

[41] Sani S., Massie S., Wiratunga N., Cooper K. (2017). Learning Deep and Shallow Features for Human Activity Recognition.

[42] Wang J., Chen Y., Hao S., Peng X., Hu L. (2019). Deep learning for sensor-based activity recognition: A survey. Pattern Recognit. Lett..

[43] Kautz T., Groh B.H., Hannink J., Jensen U., Strubberg H., Eskofier B.M. (2017). Activity recognition in beach volleyball using a Deep Convolutional Neural Network. Data Min. Knowl. Discov..

[44] LeCun Y., Bengio Y., Hinton G. (2015). Deep learning. Nature.

[45] Kingma D.P., Ba J. (2014). Adam: A method for stochastic optimisation. arXiv.

[46] Gholamiangonabadi D., Kiselov N., Grolinger K. (2020). Deep neural networks for human activity recognition with wearable sensors: Leave-one-subject-out cross-validation for model. selection. IEEE Access.

[47] Deep S., Zheng X. Leveraging CNN and Transfer Learning for Vision-based Human Activity Recognition. Proceedings of the 2019 29th International Telecommunication Networks and Applications Conference (ITNAC).

[48] Nguyen H., Lebel K., Bogard S., Goubault E., Boissy P., Duval C. (2018). Using inertial sensors to automatically detect. and segment activities of daily living in people with Parkinson’s disease. IEEE Trans. Neural Syst. Rehabil. Eng..

[49] Janidarmian M., Roshan Fekr A., Radecka K., Zilic Z. (2017). A comprehensive analysis on wearable acceleration sensors in human activity recognition. Sensors.

[50] Kowalski K., Rhodes R., Naylor P.-J., Tuokko H., MacDonald S. (2012). Direct and indirect measurement of physical activity in older adults: A systematic review of the literature. Int. J. Behav. Nutr. Phys. Act..

[51] Jasper L., Beaupre L.A., Spence J.C., Jones C.A. (2021). Validity of tools to measure physical activity in older adults following total knee arthroplasty. J. Aging Phys. Act..

[52] Lee J., Joo H., Lee J., Chee Y. (2020). Automatic classification of squat posture using inertial sensors: Deep learning approach. Sensors.

